# Blocking group 2 innate lymphoid cell activation and macrophage M2 polarization: potential therapeutic mechanisms in ovalbumin-induced allergic asthma by calycosin

**DOI:** 10.1186/s40360-024-00751-9

**Published:** 2024-04-22

**Authors:** Chunyan Tian, Qi Liu, Xiaoyu Zhang, Zhuying Li

**Affiliations:** 1 Department of Respiratory Medicine, First Affiliated Hospital, Heilongjiang University of Chinese Medicine, Harbin, China; 2 Department of Graduate, Heilongjiang University of Chinese Medicine, Harbin, China

**Keywords:** Asthma, Calycosin, Group 2 innate lymphoid cells, Macrophages

## Abstract

**Background:**

Calycosin, a flavonoid compound extracted from *Astragalus membranaceus*, has shown anti-asthma benefits in house dust mite-induced asthma. Recent studies have suggested that innate-type cells, including group 2 innate lymphoid cells (ILC2s) and macrophages, serve as incentives for type 2 immunity and targets for drug development in asthma. This work focuses on the effects of calycosin on the dysregulated ILC2s and macrophages in allergic asthma.

**Methods:**

In vivo, the asthmatic mouse model was established with ovalbumin (OVA) sensitization and challenge, and calycosin was intraperitoneally administered at doses of 20 and 40 mg/kg. In vivo, mouse primary ILC2s were stimulated with interleukin (IL)-33 and mouse RAW264.7 macrophages were stimulated with IL-4 and IL-13 to establish the cell models. Cells were treated with calycosin at doses of 5 and 10 µM.

**Results:**

In vivo, we observed significantly reduced numbers of eosinophils, neutrophils, monocyte macrophages and lymphocytes in the bronchoalveolar lavage fluid (BALF) of OVA-exposed mice with 40 mg/kg calycosin. Histopathological assessment showed that calycosin inhibited the airway inflammation and remodeling caused by OVA. Calycosin markedly decreased the up-regulated IL-4, IL-5, IL-13, IL-33, and suppression tumorigenicity 2 (ST2) induced by OVA in BALF and/or lung tissues of asthmatic mice. Calycosin repressed the augment of arginase 1 (ARG1), IL-10, chitinase-like 3 (YM1) and mannose receptor C-type 1 (MRC1) levels in the lung tissues of asthmatic mice. In vivo, calycosin inhibited the IL-33-induced activation as well as the increase of IL-4, IL-5, IL-13 and ST2 in ILC2s. Calycosin also repressed the increase of ARG1, IL-10, YM1 and MRC1 induced by IL-4 and IL-13 in RAW264.7 macrophages. In addition, we found that these changes were more significant in 40 mg/kg calycosin treatment than 20 mg/kg calycosin.

**Conclusions:**

Collectively, this study showed that calycosin might attenuate OVA-induced airway inflammation and remodeling in asthmatic mice via preventing ILC2 activation and macrophage M2 polarization. Our study might contribute to further study of asthmatic therapy.

**Supplementary Information:**

The online version contains supplementary material available at 10.1186/s40360-024-00751-9.

## Introduction

Asthma is a common respiratory disease characterized by chronic airway inflammation, bronchial hyper-responsiveness, excessive mucus secretion, and airway remodeling [1–3]. Clinically, dyspnea, wheeze, cough and chest tightness often occur in patients with asthma [1]. In addition, asthma causes severe economic and family burdens due to the high cost of treatment [4]. Therefore, it is absolutely imperative that effective drugs for asthma and the underlying mechanisms are further explored and clarified. Based on the previous immunological studies of asthma, the endotypes of asthma are divided into type 2 immune responses and non-type 2 immune responses [5, 6]. The type 2 immune response consists of T helper 2 (Th2) cells and group 2 innate lymphoid cells (ILC2s). In the study, we applied ILC2s to explore the mechanisms of calycosin for asthma treatment.

ILC2s belong to the family of innate lymphoid cells (ILCs), which are effector cells lacking T cell and B cell antigen receptors and involved in tissue remodeling and innate immunity [7]. ILCs have been classified into ILC1s, ILC2s and ILC3s on the basis of the function of cytokine production upon activation [8]. ILC2s have been extensively applied in the research associated with asthma [9, 10]. Multiple studies have stated that ILC2s are elevated in the blood of asthmatic patients [11–13]. Epithelial cell-derived cytokines, such as interleukin (IL)-25 and IL-33, are capable of stimulating ILC2s to produce T-helper (Th) type 2 cytokines, including IL-4, IL-5 and IL-13, thereby facilitating the pathogenesis of asthma [14–18]. The concentrations of IL-33 are increased in the bronchoalveolar lavage fluid (BALF) of asthmatic patients, suggesting that IL-33 is closely correlated with asthmatic severity [14, 19, 20]. Suppression tumorigenicity 2 (ST2) is known as the receptor of IL-33 and has a high expression in macrophages, natural killer cells, and ILC2s [21, 22]. A CpG-oligodeoxynucleotide alleviates Th2/Th17 inflammatory response, airway inflammation and remodeling in mice with smoke-related asthma by downregulating the IL-33/ST2 axis [23]. IL-33/ST2 signaling results in the development of airway hyper-responsiveness in the peripheral lung in house dust mite-induced asthmatic mice [24]. Therefore, targeting the inhibition of ILC2 activation by the IL-33/ST2 pathway prevents asthma, which is of great significance for studying therapeutic strategies for asthma.

The development of alternatively activated M2 macrophages is capable of being driven by ILC2s [25]. Macrophages are innate immune cells that participate in initiating an immune response [26]. Macrophages are differentiated into classically activated M1 or alternatively activated M2 macrophages [27]. Increased M2-phenotype macrophages are found in BALF and airway wall tissues in bronchial asthmatic patients [28–30]. MicroRNA-21 antagonism decreases airway hyperresponsiveness and airway remodeling process by suppressing alveolar M2 macrophage polarization in an ovalbumin (OVA)-induced allergic asthma mouse [31]. Suppression of M2 macrophage polarization by 2-chloroadenosine is accompanied by the reduction in airway inflammation and remodeling in asthmatic mice [32]. These findings indicate that drug intervention targeting the inhibition of M2 macrophage polarization may be an effective approach for asthmatic treatment.

Overall, it is vital for asthma treatment to explore novel and effective drugs that target the inhibition of ILC2 activation and M2 macrophage polarization. A previous study has shown that calycosin mitigates pulmonary inflammation and hyper-responsiveness by protecting epithelial integrity via regulating the G protein-coupled estrogen receptor in a house dust mite-induced mouse model of allergic asthma [33]. Calycosin (7,3’dihydroxy-4’-methoxyisoflavone) is a flavonoid component of *Astragalus membranaceus*, which has a great effect on the treatment of the recurrence of allergic diseases [33, 34]. Calycosin has been proven to possess the properties of immune regulation and anti-inflammation [35]. Calycosin ameliorates renal injury in diabetic rats by inhibiting of the mRNA expression of both IL-33 and ST2 [36]. Hence, we conjectured that calycosin might be a potential targeted medicine for asthma by regulating ILC2 activation and M2 macrophage polarization.

Herein, an OVA-sensitized asthmatic mouse model was established to investigate the effects of calycosin. It was investigated that calycosin repressed ILC2 activation and macrophage M2 polarization, thereby ameliorating asthma.

## Materials and methods

### Animal model

The animal model was established following previous literature [37]. Female BALB/C mice (6–8 weeks) were obtained from Liaoning Changsheng Biotechnology Company Limited (China). For establishing a mouse model of asthma, the mice were divided into four groups, including sham, OVA, OVA + 20 mg/kg calycosin (OVA + 20Ca), and OVA + 40 mg/kg calycosin (OVA + 40Ca) groups. The mice in the OVA group were intraperitoneally injected with 100 µl phosphate-buffered saline (PBS) containing 100 µg OVA (A107820, Aladdin, Shanghai, China) and 4 mg aluminum hydroxide on days 0, 7, and 14. On days 21 to 26, the mice were treated with aerosolized 1% OVA for 30 min each day. 20 and 40 mg/kg calycosin (≥ 98% purity, verified by High Performance Liquid Chromatography (HPLC), C_16_H_12_O_5_; B20846, Yuanye, Shanghai, China) were administered to the mice in the OVA + 20Ca and OVA + 40Ca groups respectively by intraperitoneal injection at 1 h before atomization. The doses were selected because of the potential effectiveness in asthma according to the previous literatures [38–40]. The mice in the sham group were given the same volume of PBS without OVA. Twenty-four hours after the last OVA challenge, the mice were euthanized by isoflurane inhalation (3% for induction and 1.5% for maintenance) and exsanguination from the inferior vena cava. Serum was collected. The lung tissues were stored at -70°C or fixed in 4% paraformaldehyde for further experiments. All animal experiments were approved by the Ethics Committee of the First Affiliated Hospital of Heilongjiang University of Chinese Medicine (2022062703). All methods were conducted according to the relevant guidelines and regulations for handling laboratory animals.

### Cell isolation and detection

ILC2s were isolated from the lung tissues according to the previous study [41]. In short, the lung tissues were filtered via a 40-µm cell strainer and placed in a six-well culture dish with 7 ml RPMI-1640 medium (31800, Solarbio, Beijing, China) containing 50 µg/ml Liberase TM (5401119001, Sigma, USA) and 1 µg/ml DNase I (BS137, Biosharp, Hefei, China). After treatment with red blood lysis (C3702, Beyotime, Shanghai, China), the samples were fractionated with Percoll (BS909, Biosharp, Hefei, China). The acquired cells were incubated with PE anti-mouse cluster of differentiation (CD)45 (clone 30-F11) antibody (F2104502, Multisciences Biotech, Hangzhou, China), FITC anti-mouse CD25 antibody (FITC-65137, Proteintech, Wuhan, China), PE anti-mouse ST2 (clone RMST2-2) antibody (12-9335-80, ThermoFisher Scientific, USA) and PE anti-mouse receptor tyrosine kinase (c-Kit) antibody (161605, Biolegend, USA) at 4 °C for 30 min in the dark. Ultimately, ILC2s were isolated and collected by gating strategies (gated as live CD45 + CD25 + ST2 + c-Kit + cells) using flow cytometry (NovoCyte, Agilent, USA).

### Cell culture and treatment

The ILC2s were cultured in the RPMI-1640 medium (31800, Solarbio, Beijing, China) containing IL-2 (10 ng/ml; 212 − 12, PeproTech, USA) and 10% fetal bovine serum (FBS; 11011 − 8611, Solelybio, Hangzhou, China) [41]. ILC2s were treated with calycosin (5 or 10 µM; B20846, Yuanye, Shanghai, China) after IL-33 (100 ng/ml; 210 − 33, PeproTech, USA) [42] for 48 h at 37°C with 5% CO_2_.

Mouse RAW264.7 macrophages were purchased from iCell Bioscience Inc. (Shanghai, China) and maintained in DMEM medium (G4510, Servicebio, Wuhan, China) with 10% FBS. RAW264.7 cells were subjected to IL-4 (10 ng/ml; 51,084-MNAE, Sino Biological, Beijing, China) and IL-13 (10 ng/ml; Z03191, GenScript, Nanjing, China) [43], while being treated with calycosin (5 or 10 µM) for 48 h [44].

### Cell counts in the BALF

BALF was harvested from the mice. The total inflammatory cell counts including eosinophils, neutrophils, monocyte macrophages and lymphocytes were determined in the BALF by Giemsa (D010, Jiancheng Bioengineering Institute, Nanjing, China) staining.

### Enzyme-linked immunosorbent assay (ELISA)

The levels of immunoglobulin E (IgE) in serum were examined on the basis of the Mouse IgE ELISA kit (Multisciences Biotech, Hangzhou, China). The levels of IL-4, IL-5, IL-13, IL-33 and IL-10 in the BALF or the cells were detected using the corresponding Mouse IL-4/IL-5/IL-13/IL-33/IL-10 ELISA Kit (Multisciences Biotech, Hangzhou, China). The concentrations of arginase 1 (ARG1) in the BALF or RAW264.7 cells were detected by the Mouse ARG1 ELISA kit from Fine Biotech (Wuhan, China).

### Hematoxylin-eosin (H&E) and periodic acid-Schiff (PAS)

Pulmonary morphology was analyzed through H&E and PAS staining [45]. Briefly, the fixed lung tissues were embedded in paraffin and cut into 5 μm-thick sections, which were stained by hematoxylin (H8070, Solarbio, Beijing, China) and eosin (A600190, Sangon, Shanghai, China). The degree of inflammatory cell infiltration was scored according to the previous study [46].

Additionally, PAS (DG0005, Leagene, Beijing, China) was used to stain the slices. The images were captured and observed at 200 × magnification by a microscope (Olympus, Tokyo, Japan). The degree of airway wall thickening and mucus hypersecretion was scored according to the previous study [47].

### Reverse transcription-quantitative PCR (RT-qPCR)

Total RNA from the lung tissues and RAW264.7 cells was abstracted by TRIpure lysis (RP1001, BioTeke, Beijing, China). The cDNA was generated with BeyoRT II M-MLV Reverse Transcriptase (D7160L, Beyotime, Shanghai, China). The relative expression of mRNA was evaluated by RT-qPCR using SYBR Green (SY1020, Solarbio, Beijing, China) in Exicycler™ 96 Real-Time Quantitative Thermal Block (Bioneer, Daejeon, Korea). The specific primers were as follows: IL-4, 5’-CATCCTGCTCTTCTTTCTCG-3’, 5’-CCTTCTCCTGTGACCTCGTT-3’; IL-5, 5’-GGCTTCCTGTCCCTACT-3’, 5’-CTTCCATTGCCCACTCT-3’; IL-13, 5’-TTGCCTTGGTGGTCTCG-3’, 5’-CAATATCCTCTGGGTCCTGT-3’; chitinase-like 3 (YM1), 5’-ACTGGAATTGGTGCCCCTAC-3’, 5’-GAGCCACTGAGCCTTCAACT-3’; mannose receptor C-type 1 (MRC1), 5’-AGTGATGGTTCTCCCGTTTC-3’, 5’-TGGGCTCAGGTAGTAGTGTTTT-3’. GAPDH was used as an endogenous control.

### Immunohistochemistry (IHC)

Antigen retrieval was performed in the lung slices after dewaxing and rehydration, followed by incubation with 3% H_2_O_2_ (10011218, Sinoreagent, Shanghai, China). The samples were blocked in 1% bovine serum albumin (BSA; A602440-0050, Sangon, Shanghai, China) for 15 min. The sections were incubated with ST2 antibody (1:50; 11920-1-AP, ProteinTech, Wuhan, China) at 4°C overnight and subjected to HRP-labeled goat anti-rabbit IgG (1:500; # 31460, ThermoFisher Scientific, USA) at 37°C for 1 h. Additionally, IL-25R expression was detected in the same way. The sections were incubated with IL-25R antibody (1:100; PA5-47051, ThermoFisher Scientific, USA) at 4°C overnight and next incubated with HRP-labeled donkey anti-goat IgG (1:500; ab6885, Abcam, Cambridge, UK) at 37°C for 1 h. Subsequently, these pieces were stained with 3, 3-diaminobenzidine tetrahydrochloride (DAB; DAB-1031, Maixin, Fuzhou, China) and counterstained with hematoxylin (H8070, Solarbio, Beijing, China). The results were observed under a microscope (Olympus, Tokyo, Japan) at 400 × magnification.

### Immunofluorescence (IF)

IF was used to determine the co-localization of ST2 and CD45 to show the levels of double-positive ILC2s (ST2^+^CD45^+^) in the lung tissues [48]. Besides, the co-localization of EGF-like module-containing mucin-like hormone receptor-like 1 (F4/80) and MRC1 in the lung tissues was detected to show the levels of double-positive M2 macrophages (F4/80 + MRC1+) using IF [49]. The lung slices were dewaxed, rehydrated and restored antigen, and then they were blocked in 1% BSA. The tissue samples were incubated with anti-rabbit ST2 (1:100; 11920-1-AP, ProteinTech, Wuhan, China), anti-mouse CD45 (1:50; Sc-19,615, Santa Cruz Biotechnology, USA), anti-rabbit MRC1 (1:100; DF4149, Affinity Biosciences, Changzhou, China) or anti-mouse F4/80 (1:50; sc-377009, Santa Cruz Biotechnology, USA) antibodies at 4°C overnight. Subsequently, the samples were probed with Cy3-conjugated goat anti-rabbit IgG (1:200; A27039, Invitrogen, USA) or FITC-labeled goat anti-mouse IgG (1:200; ab6785, Abcam, Cambridge, UK) secondary antibodies at room temperature for 90 min. The images were captured with a microscope (Olympus, Tokyo, Japan) at 400 × magnification.

Similarly, we examined MRC1 expression in RAW264.7 cells by IF assay. RAW264.7 cells were fixed in 4% paraformaldehyde (80096618, Sinoreagent, Shanghai, China) for 15 min and permeated in 0.1% Triton X-100 (ST795, Beyotime, Shanghai, China) for 30 min. After being covered with 1% BSA for 15 min, the cells were exposed to the MRC1 (1:100; DF4149, Affinity Biosciences, Changzhou, China) antibody at 4°C overnight. The corresponding second antibody, Cy3-conjugated goat anti-rabbit IgG (1:200; A27039, Invitrogen, USA), was added and treated for 1 h. The tissues or cells were stained with 4’, 6-diamidino-2-phenylindole (DAPI; D106471, Aladdin, Shanghai, China). Finally, the images were gathered under a microscope (Olympus, Tokyo, Japan) at 400 × magnification.

### Cell counting kit 8 (CCK-8)

The ILC2s were seeded in 96-well plates (8 × 10^3^ cells per well) and cultured at 37°C for 48 h. Next, 10 µl CCK-8 (KGA317, KeyGene Biotech, Nanjing, China) was added into each well to develop for 2 h. A microplate reader (800TS, BioTek, USA) was applied to measure the OD values at 450 nm.

### Western blot

The lung tissues and ILC2s were lysed with radio immunoprecipitation assay lysis buffer (R0010, Solarbio, Beijing, China) on ice. Total protein was quantified via the BCA Protein Assay Kit (PC0020, Solarbio, Beijing, China). The protein was resolved using sodium dodecyl sulfate-polyacrylamide gel electrophoresis (SDS-PAGE; D1010, Solarbio, Beijing, China). The samples were transferred to polyvinylidene difluoride membranes (IPVH00010, Millipore, USA). Due to the stable positions of the target protein blots and in order to obtain the clearer bands, all the membranes were cropped prior to hybridization with primary antibodies. Then, they were blocked with 5% nonfat milk (A600669, Sangon, Shanghai, China). Subsequently, the membranes were incubated with anti-rabbit ST2 antibody (1:500; 11920-1-AP, ProteinTech, Wuhan, China) and anti-mouse GAPDH antibody (1:10000; 60004-1-Ig, ProteinTech, Wuhan, China) at 4°C. The next day, they were treated with the corresponding secondary antibody, HRP-labeled goat anti-rabbit IgG (1:3000; SE134, Solarbio, Beijing, China) or goat anti-mouse IgG (1:3000; SE131, Solarbio, Beijing, China) at 37°C for 1 h. The ST2 protein was visualized using ECL Western Blotting Substrate (PE0010, Solarbio, Beijing, China).

### Statistical analysis

GraphPad Prism 8.0 software was utilized to analyze all the data, which were presented as mean ± SD. A one-way ANOVA with Tukey’s test was performed for multiple group comparisons. Kruskal-Wallis non-parametric test was used to compare the pathology scores among multiple groups. Significance was set at p-value < 0.05.

## Results

### Calycosin protected asthmatic mice from inflammatory responses

**Fig. 1:**
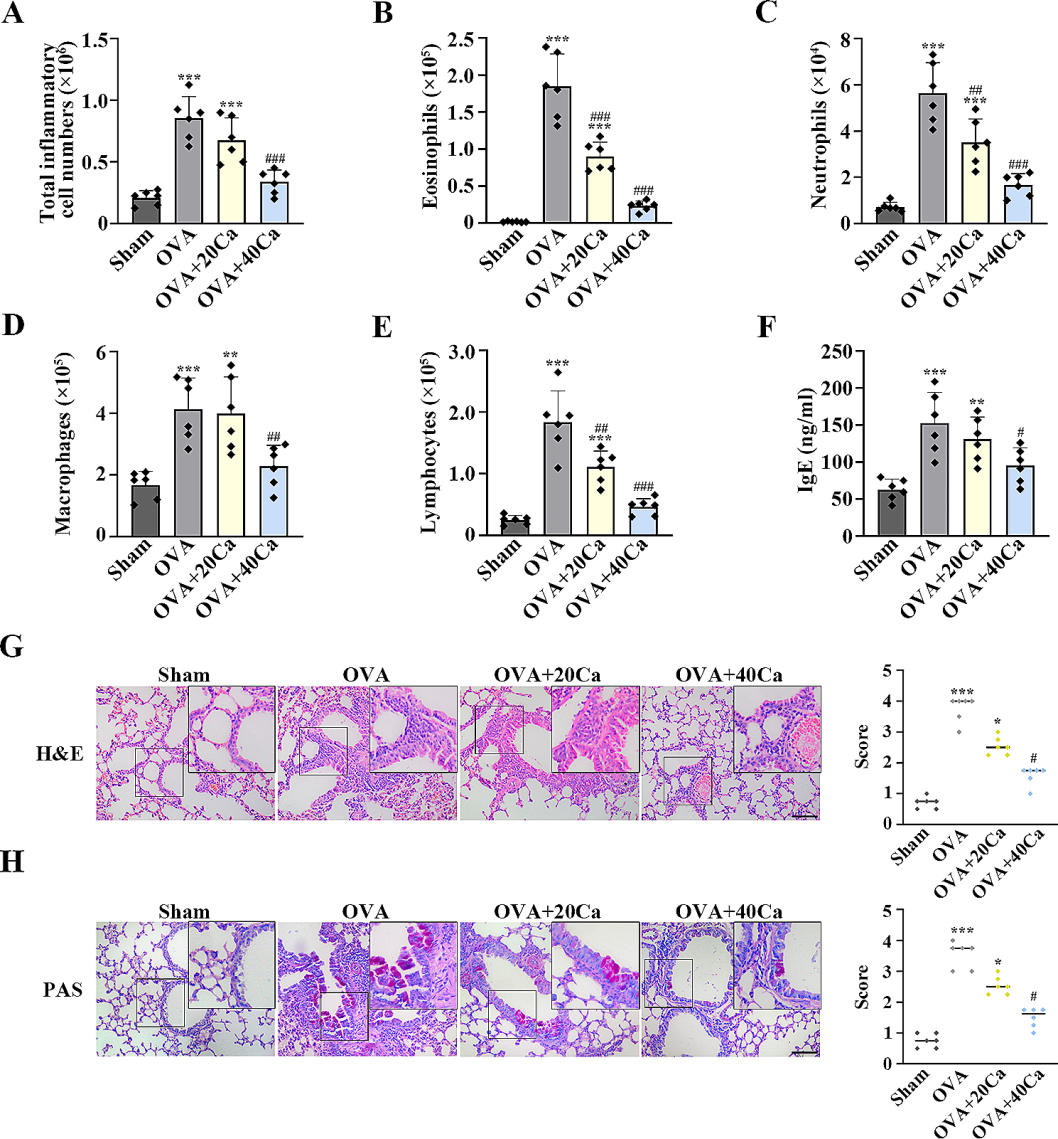
Calycosin reduced inflammation in ovalbumin (OVA)-induced asthmatic mice. (**A**) Total inflammatory cells, including (**B**) eosinophils, (**C**) neutrophils, (**D**) macrophages and (**E**) lymphocytes, were counted in the bronchoalveolar lavage fluid (BALF). (**F**) ELISA assay evaluated the levels of serum immunoglobulin E (IgE). (**G**) The lung sections were stained with Hematoxylin-Eosin (H&E). The degree of inflammatory cell infiltration was scored (0–4). (**H**) The lung sections were stained with Periodic acid-Schiff (PAS). The degree of airway wall thickening and mucus hypersecretion was scored (0–4). Scale bar = 100 μm. *n* = 6 for each group. ^*^P < 0.05, ^**^P < 0.01, ^***^P < 0.001, compared with the sham group; ^#^*P* < 0.05, ^##^*P* < 0.01, ^###^*P* < 0.001, compared with the OVA group. “Ca” represents “calycosin”

Calycosin repressed the increase of inflammatory cells, including eosinophils, neutrophils, monocyte macrophages and lymphocytes, induced by OVA in the mouse BALF (*p* < 0.01, Fig. 1A–E). The up-regulated serum IgE levels in OVA-induced asthmatic mice were suppressed by calycosin (*p* < 0.05, Fig. 1F). H&E staining showed that calycosin reduced OVA-stimulated infiltration of inflammatory cells in the peribronchial and perivascular tissues (*p* < 0.05, Fig. 1G). As shown in Fig. 1H, OVA induced airway wall thickening and mucus hypersecretion, which were mitigated by calycosin (*p* < 0.05, Fig. 1H). Administration of high-dose calycosin (40 mg/kg) displayed better effects than low-dose calycosin (20 mg/kg; Fig. 1). These findings revealed that OVA resulted in mouse inflammatory responses, which were prevented by calycosin.

### Calycosin restrained ILC2 activation in asthmatic mice

**Fig. 2:**
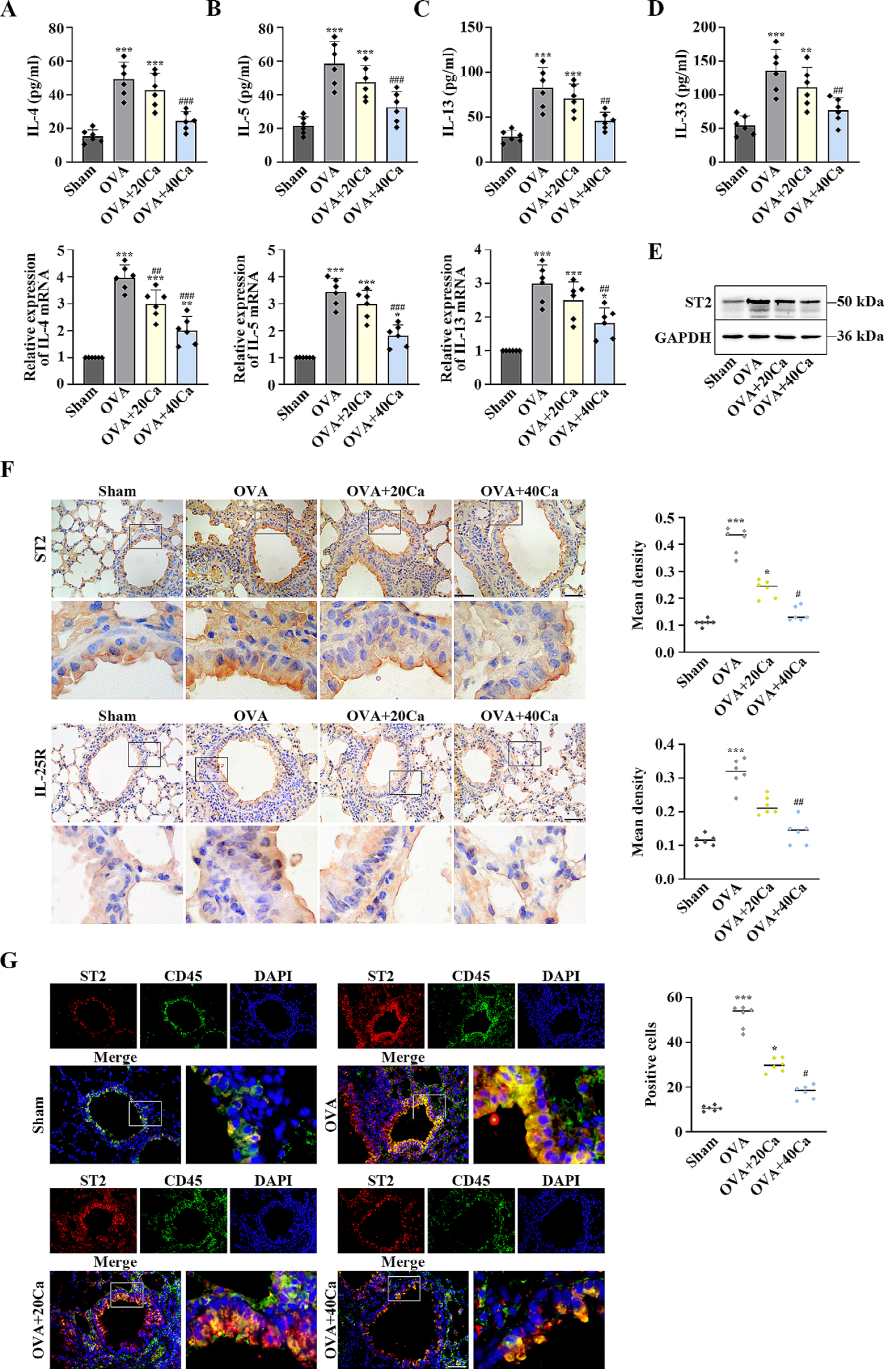
Calycosin inhibited group 2 innate lymphoid cell (ILC2) activation in OVA-induced asthmatic mice. (**A**) The levels of interleukin (IL)-4, (**B**) IL-5 and (**C**) IL-13 in the BALF and lung tissues were measured using ELISA and RT-qPCR. (**D**) ELISA was used to determin the contents of IL-33 in the BALF. (**E**) Suppression tumorigenicity 2 (ST2) levels in the lung tissues was examined by western blot. The blots were cut prior to hybridization with antibodies during blotting. (**F**) Immunohistochemistry (IHC) staining was used to detect the expression ST2 and IL-25R. (**G**) The co-localization of ST2 and CD45 was detected by immunofluorescence (IF). Scale bar = 50 μm. *n* = 6 for each group. ^*^ *P*< 0.05, ^**^P < 0.01, ^***^P < 0.001, compared with the sham group; ^#^*P* < 0.05, ^##^*P* < 0.01, ^###^*P* < 0.001, compared with the OVA group. “Ca” represents calycosin

The levels of Th2-type cytokines (IL-4, IL-5 and IL-13) were elevated in OVA-sensitized asthmatic mice compared with the mice in sham group. However, calycosin decreased the release of these cytokines compared with the mice in OVA group in the BALF and lung tissues (*p* < 0.05, Fig. 2A–C). In addition, calycosin diminished the increase of IL-33 in the BALF (*p* < 0.01, Fig. 2D). Western blot assay demonstrated that ST2 protein was highly expressed in the mice with asthma, while it was downregulated in the mice with calycosin treatment (Fig. 2E). Furthermore, calycosin ameliorated OVA-induced increases in ST2 and IL-25R levels in the lung tissues (*p* < 0.05, Fig. 2F). The co-localization of ST2 and CD45 was facilitated in OVA-induced mouse lung tissues. The co-localization was suppressed by calycosin (*p* < 0.05, Fig. 2G). Our data indicated that calycosin exerted a negative role in ILC2 activation in OVA-challenged asthmatic mice.

### Calycosin blocked macrophage M2 polarization in asthmatic mice

**Fig. 3:**
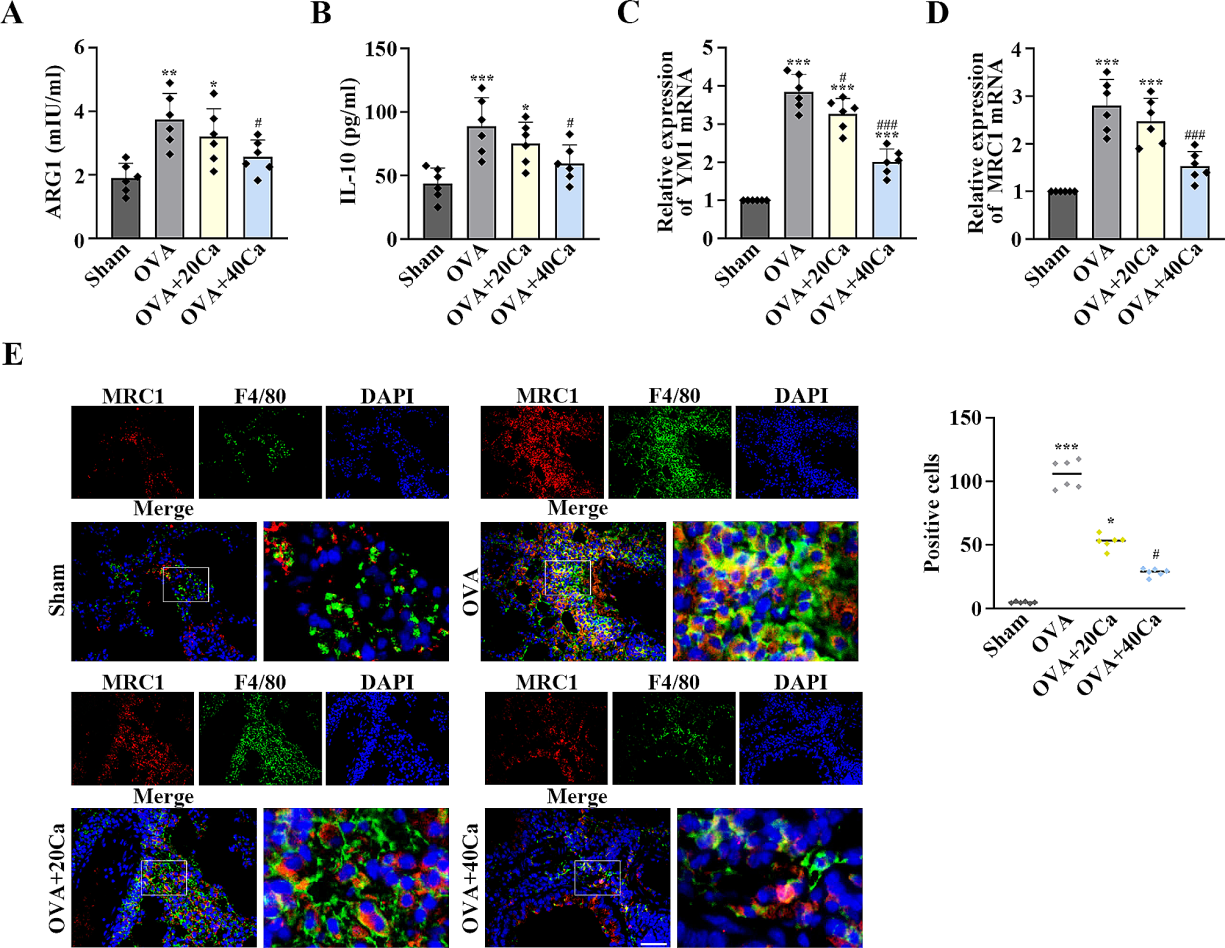
Calycosin decreased macrophage M2 polarization in OVA-induced asthmatic mice. (**A**) The levels of M2 macrophage markers arginase 1 (ARG1) and (**B**) IL-10 in the BALF and (**C**) chitinase-like 3 (YM1) and (**D**) Mannose receptor C-type 1 (MRC1) in the lung tissues were estimated by ELISA and RT-qPCR, respectively. (**E**) IF was performed to present co-localization of MRC1 and EGF-like module-containing mucin-like hormone receptor-like 1 (F4/80) in the lung tissues. Scale bar = 50 μm. *n* = 6 for each group. ^*^P < 0.05, ^**^P < 0.01, ^***^P < 0.001, compared with the sham group; ^#^*P* < 0.05, ^###^*P* < 0.001, compared with the OVA group. “Ca” represents calycosin

The protein levels of M2 macrophage markers ARG1 and IL-10 were assessed in the BALF by ELISA (Fig. 3A and B). The mRNA levels of YM1 and MRC1 were detected in the lung tissues by RT-qPCR (Fig. 3C and D). OVA led to the up-regulation of these M2 macrophage markers. However, the levels of these markers were declined after the administration of calycosin (*p* < 0.05, Fig. 3A–D). Calycosin obstructed the co-localization of MRC1 and F4/80 induced by OVA in asthmatic mice (*p* < 0.05, Fig. 3E). Therefore, calycosin attenuated macrophage M2 polarization in the mice treated with asthma.

### Calycosin inhibited IL-33-induced ILC2 activation in ILC2s

**Fig. 4:**
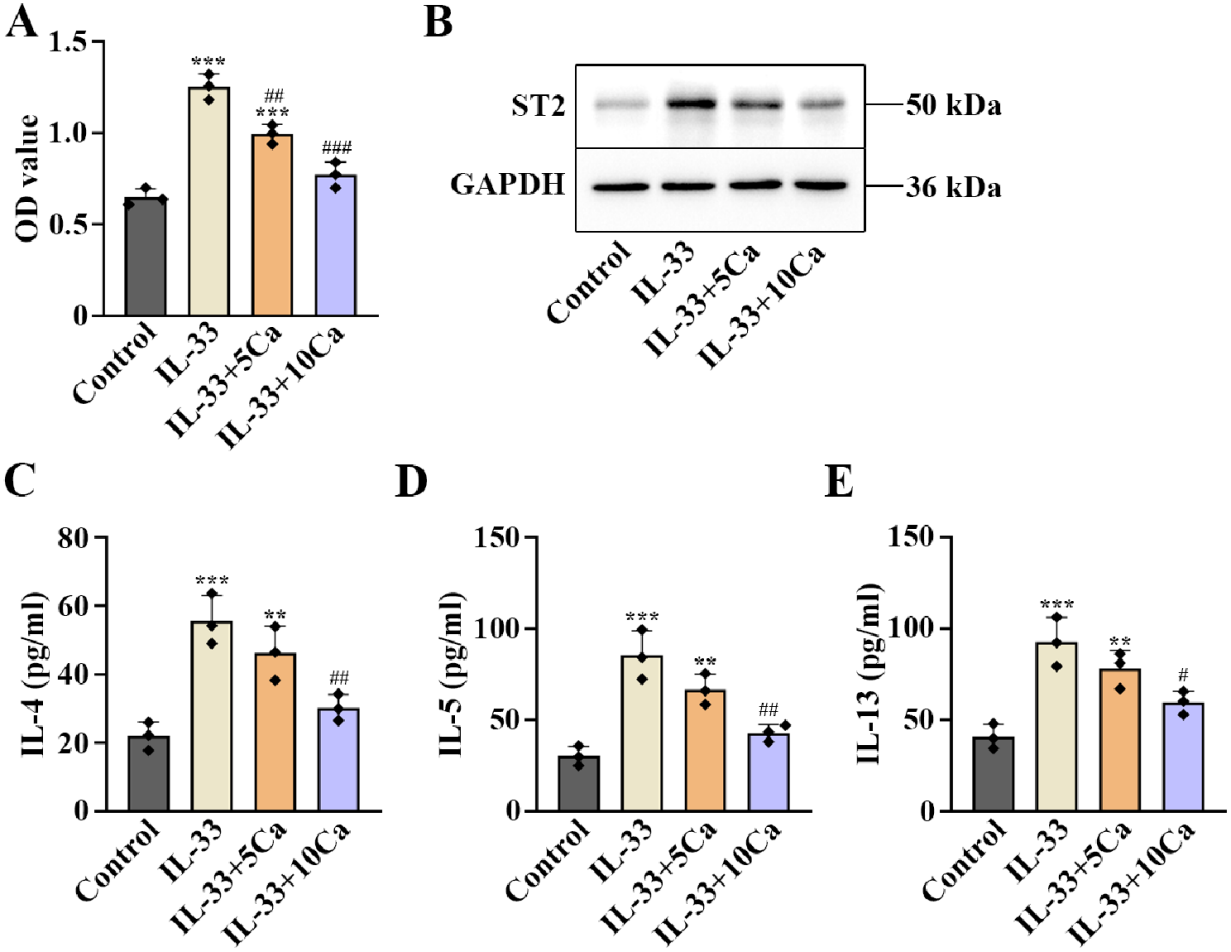
ILC2 activation induced by IL-33 was restrained by calycosin in ILC2s. (**A**) The ILC2 cell viability was examined by CCK-8. (**B**) Western blot showed the expression of ST2 in ILC2s. The blots were cut prior to hybridization with antibodies during blotting. (**C**) The secretion of IL-4, (**D**) IL-5 and (**E**) IL-13 in ILC2s were detected by ELISA assay. *n* = 3 for each group. ^**^P < 0.01,^***^P < 0.001, compared with the control group; ^#^*P* < 0.05, ^##^*P* < 0.01, ^###^*P* < 0.001, compared with the IL-33 group. “Ca” represents calycosin

To further investigate the roles of calycosin in ILC2 activation, we isolated ILC2s from the lung tissues of BALB/C mice. The ILC2s were identified by detecting the cell surface molecules, including CD45, CD25, ST2 and c-Kit (Figure S1A–D). IL-33 treatment increased the viability in the isolated ILC2s. Calycosin reversed the effects (*p* < 0.01, Fig. 4A). On the one hand, calycosin decreased the increase in ST2 protein levels induced by IL-33 in ILC2s (Fig. 4B). On the other hand, calycosin suppressed the enhanced release of IL-4, IL-5 and IL-13 in IL-33-treated ILC2s (*p* < 0.05, Fig. 4C–E). It was suggested that ILC2 activation was repressed by calycosin in ILC2s.

### Calycosin prevented IL-4 and IL-13-activated M2 polarization in RAW264.7 macrophages

**Fig. 5:**
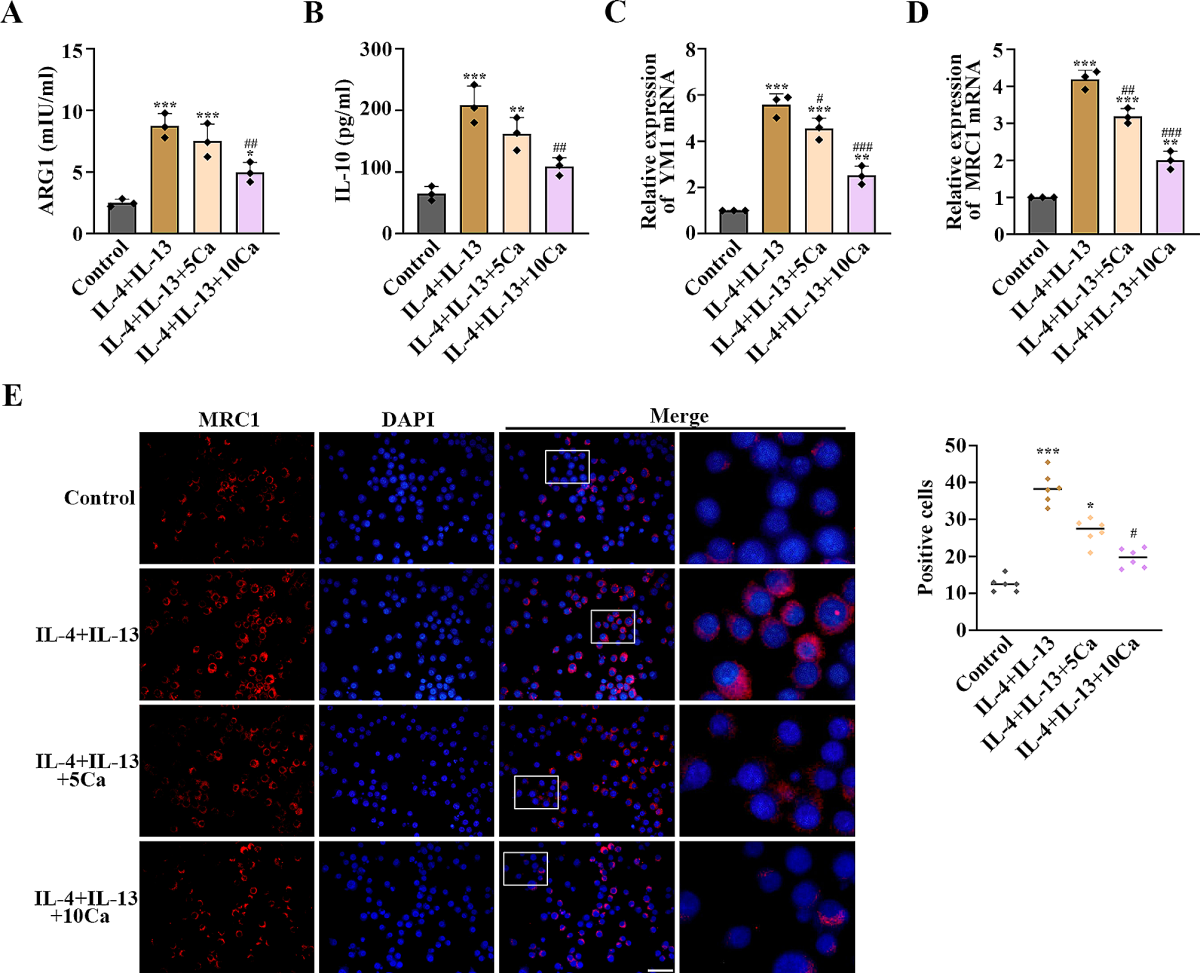
Calycosin suppressed macrophage M2 polarization caused by IL-4 and IL-13 in RAW264.7 cells. (**A**) The levels of M2 macrophage markers ARG1, (**B**) IL-10, (**C**) YM1 and (**D**) MRC1 in RAW264.7 cells were determined. (**E**) MRC1 content was visualized by IF staining. Scale bar = 50 μm. *n* = 3 for each group. ^*^P < 0.05, ^**^P < 0.01, ^***^P < 0.001, compared with the control group; ^#^*P* < 0.05, ^##^*P* < 0.01, ^###^*P* < 0.001, compared with the IL-4 + IL-13 group. “Ca” represents calycosin

The levels of ARG1, IL-10, YM1, and MRC1 were increased in RAW264.7 macrophages after the treatment of IL-4 and IL-13 compared with the control cells, and then declined after administering calycosin (*p* < 0.05, Fig. 5A–D). Calycosin reduced the increase of MRC1 levels induced by IL-4 and IL-13 in RAW264.7 cells (*p* < 0.05, Fig. 5E). It was implied that calycosin was able to block IL-4 and IL-13-activated polarization of RAW264.7 macrophages to an M2 phenotype.

## Discussions

In this study, we established an asthmatic mouse model using OVA. Calycosin inhibited the number of inflammatory cells in asthmatic mice. Additionally, calycosin exerted inhibitory effects on the enhanced inflammatory responses that were caused by OVA in mice. Besides, we elucidated that calycosin markedly alleviated OVA-challenged asthma in mice through obstructing ILC2 activation and macrophage M2 polarization via measuring the associated markers.

ILC2s, corresponding to Th2 cells in adaptive immunity, have been confirmed to play a dominant role in allergic diseases such as asthma [50]. The patients with asthma have more ILC2s in the blood and sputum [12, 13, 51]. IL-33, which serves as a Th2 cytokine activator in ILC2s, is closely involved in inflammatory conditions [52]. When subjects with asthma have been challenged with an inhalational allergen, IL-33 is ascending in the bronchial epithelium [53, 54]. IL-33 activates lung ILC2s by binding its receptor ST2, which is highly expressed in ILC2s in asthmatic mice [55, 56]. It has been discovered that the IL-33/ST2 pathway plays a significant role in asthma [57]. In the previous study, up-regulated IL-33 and ST2 expression in OVA-sensitized lungs are reduced through salidroside administration, reflecting that salidroside treats allergic asthma by repressing lung ILC2 activation via targeting the IL-33/ST2 axis [45]. Osthole attenuates OVA-caused lung inflammation via blocking IL-33/ST2 signaling in asthmatic mice [58]. In line with these studies above, the levels of IL-33 and ST2 were both up-regulated in the lung tissues in OVA-exposed asthmatic mice. However, we found that calycosin downregulated the levels of IL-33, and ST2 to diminish ILC2 activation induced by OVA in mice. In addition, calycosin inhibited IL-33-induced the increase of ST2 to suppress ILC2 activation in vitro. These results indicated that calycosin targeted the IL-33/ST2 axis to suppress ILC2 activation, thereby serving as a protective medicine in asthma.

IL-33 administration stimulates ILC2s to produce IL-4 and IL-13, which are capable of polarizing M2 macrophages [59–61]. More and more researchers have observed that M2 macrophage polarization is closely linked with the development of asthma [28, 29, 62]. Moreira and colleagues have discovered that serum amyloid P prevents airway resistance to methacholine, inflammation, and remodeling in asthmatic mice with *Aspergillus fumigatus* allergy, which may involve the inhibition of the M2 activation of macrophages [63]. Decreased M2 macrophage polarization suppresses asthmatic airway inflammation in the microRNA-21-deficiency lung tissues in OVA-induced mice [31]. Histone deacetylase 8 inhibitor ameliorates airway hyper-responsiveness and allergic airway inflammation in mice with allergic asthma through declining M2 macrophage polarization [64]. These studies have suggested that inhibiting M2 macrophage polarization is significant for asthma treatment. In our study, the levels of ARG1, IL-10, YM1, MRC1 and F4/80 were used to reflect the levels of M2-polarized macrophages according to the previous literatures [65–69]. Our study manifested that calycosin blocked OVA-induced M2 macrophage polarization in mice and repressed M2 macrophage polarization driven by IL-4 and IL-13 in RAW264.7 macrophages. It was thus clear that the protective role of calycosin in asthma might be due to the suppression of M2 macrophage polarization.

Calycosin is a typical phytoestrogen extracted from the root of *Astragalus membranaceus* that is effective in treating cardiovascular disease, nephritis, diabetes mellitus, childhood asthma, allergic rhinitis and cancers [39]. Calycosin has been reported to possess multiple biological activities, such as anti-oxidative stress, anti-inflammatory, antibacterial, and neuroprotective effects [39]. Calycosin reduces diabetes-induced kidney inflammation through inhibiting phosphorylation of p65 in the nuclear factor-κB (NF-κB) signaling pathway [70, 71]. Calycosin suppresses autophagy and oxidative stress in chronic kidney disease skeletal muscle atrophy by regulating the AMPK/SKP2/CARM1 signaling pathway [72]. Interestingly, previous studies have shown that calycosin suppresses IL-33 levels in various inflammatory disorders [36, 73]. Calycosin significantly attenuates renal injury in diabetic rats, involving inhibition of IL-33/ST2 axis signaling [36]. In our study, calycosin was capable of reducing the levels of IL-33 and ST2 to inhibit ILC2 activation in asthma. Currently, it has not been reported that ILC2 activation and M2 macrophage polarization are involved in asthma therapy by calycosin. Our research might provide new insights for the treatment of asthma.

This study has the following limitations: Firstly, although we preliminarily investigated that calycosin might block ILC2 activation and M2 macrophage polarization to alleviate asthma in mice, there are no further in vivo experiments to prove the toxicity of calycosin on other cells. Thus, it is still necessary to further determine whether calycosin safely exerts its therapeutic effects in vivo. Secondly, the current researches have suggested that calycosin is almost nontoxic. However, excessively low or high doses may cause side effects in patients. It is of great significance to explore the optimal concentration of calycosin clinically in the future. Thirdly, although we found that the cytokines or markers, such as IL-33, ST2, IL-4 and MRC1, participated in the treatment of calycosin in asthma, the direct molecular mechanism of action is still unclear. We may use molecular docking and single cell RNA sequencing (scRNA-seq) to further investigate the potential molecular mechanism in asthma in the future. Fourthly, it is one-sided that RAW264.7 cells are used instead of primary cells. Although RAW264.7 cells have been directly applied to study the roles of macrophages in multiple studies, it is still necessary to verify the results in primary cells in the future for more rigorous and credible results. Finally, in vivo experiments should be further improved. We used IF to determine the expression of ST2 and CD45 to show the levels of ILC2s (ST2 + CD45+) and F4/80 and MRC1 to show the levels of M2 macrophages (F4/80 + MRC1+) in mouse lung tissues. However, it is significant to further investigate the number of ILC2 and M2 macrophages in fresh BALF and the phenotype changes in these two cell types after treatment with calycosin.

## Conclusions

In summary, we found that calycosin protected asthmatic mice from inflammation injury by targeting the suppression of ILC2 activation and macrophage M2 polarization. Our study might provide a promising strategy for asthma therapy.

### Electronic supplementary material

Below is the link to the electronic supplementary material.

Supplementary Material 1

Supplementary Material 2

## Data Availability

The datasets used and/or analyzed during the current study are available from the corresponding author on reasonable request.

## Abbreviations

ILC2s: group 2 innate lymphoid cells
OVA: ovalbumin
IL: interleukin
BALF: bronchoalveolar lavage fluid
ST2: Suppression tumorigenicity 2
ARG1: arginase 1
YM1: chitinase-like 3
MRC1: mannose receptor C-type 1
Th: T-helper type
PBS: phosphate-buffered saline
FBS: fetal bovine serum
ELISA: Enzyme-linked immunosorbent assay
IgE: immunoglobulin E
H&E: Hematoxylin-Eosin
PAS: Periodic acid-Schiff
RT-PCR: Reverse transcription-quantitative PCR
IHC: Immunohistochemistry
BSA: bovine serum albumin
DAB: 3, 3-diaminobenzidine tetrahydrochloride
IF: Immunofluorescence
DAPI: 4’, 6-diamidino-2-phenylindole
CCK-8: Cell counting kit 8
SDS-PAGE: sodium dodecyl sulfate-polyacrylamide gel electrophoresis
