# Supplementary figures and images for: Blocking group 2 innate lymphoid cell activation and macrophage M2 polarization: potential therapeutic mechanisms in ovalbumin-induced allergic asthma by calycosin
Source: BMC Pharmacol Toxicol. 2024 Apr 22;25:30. doi: 10.1186/s40360-024-00751-9 (PMC11036756; doi:10.1186/s40360-024-00751-9)

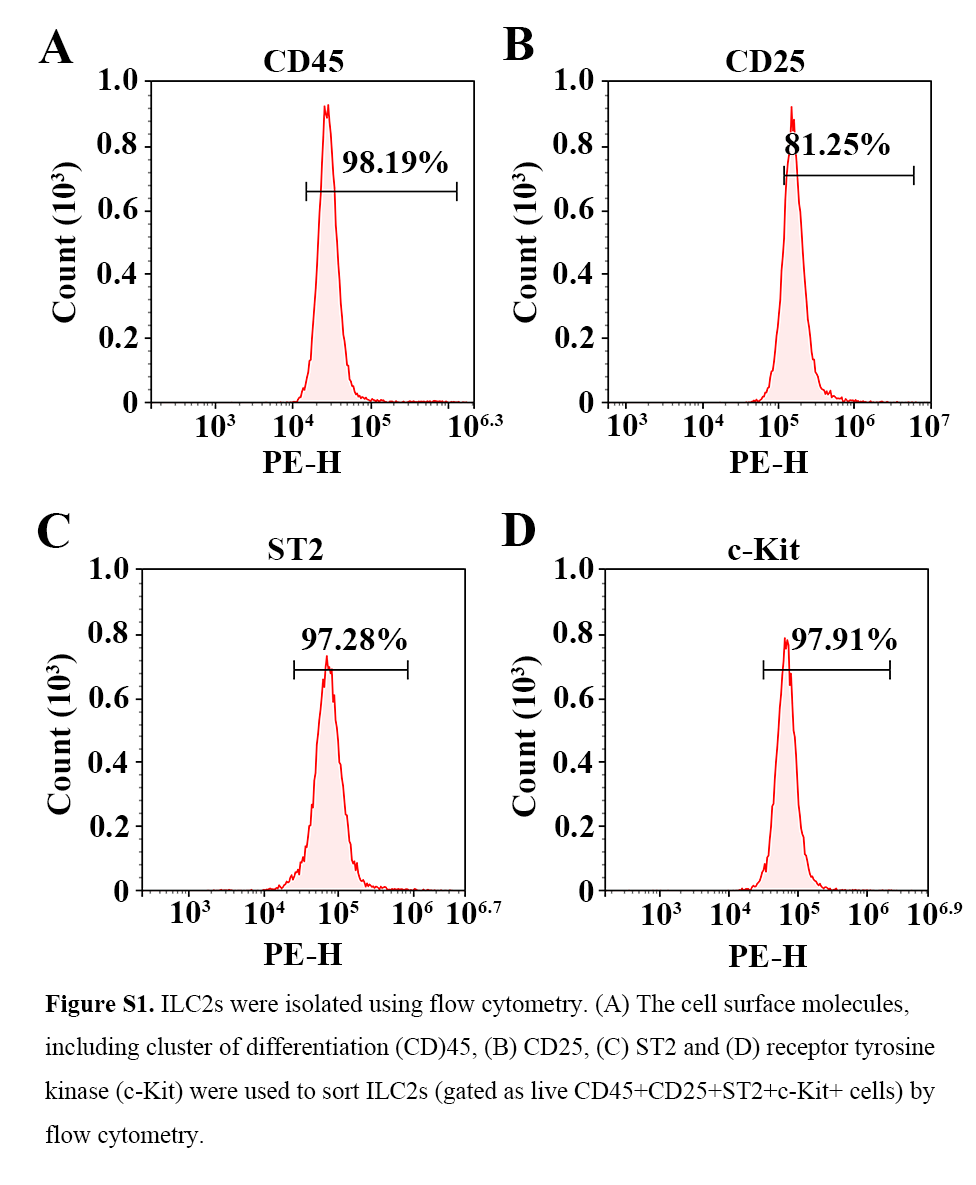

Supplement: Supplementary file 1 — Supplementary Material 1 [file 40360_2024_751_MOESM1_ESM.tif]

Fig.2E

Repeat 1

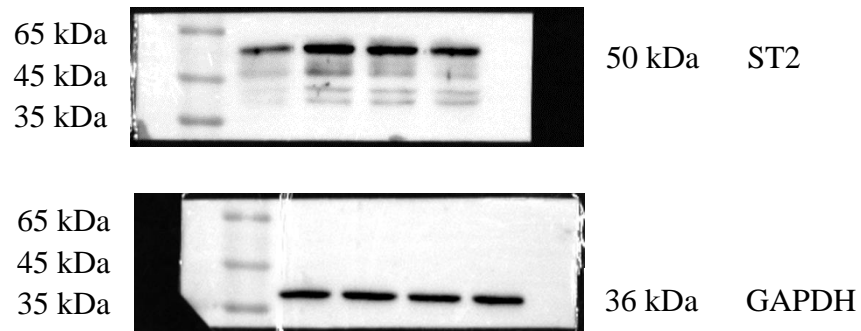

Repeat 2

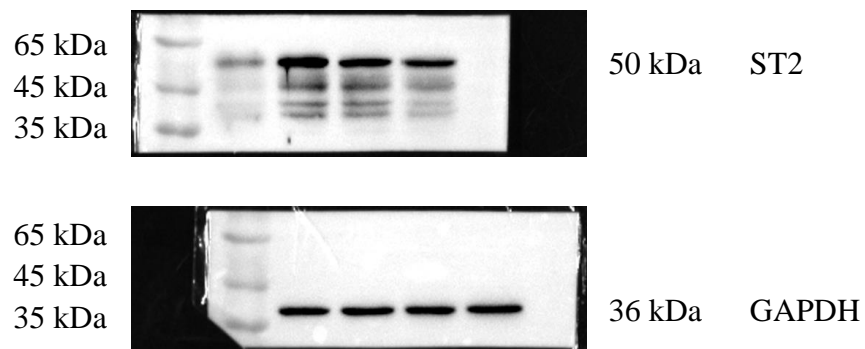

Repeat 3

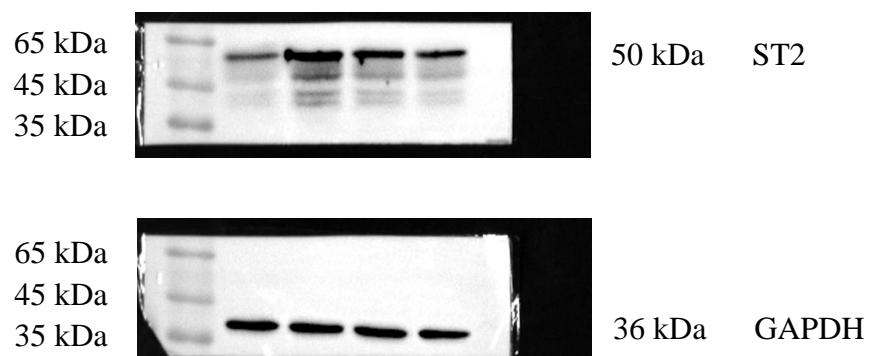

Fig.4B

Repeat 1

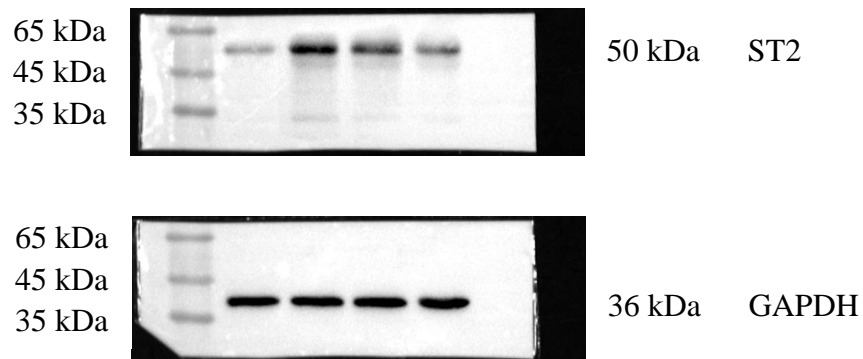

Repeat 2

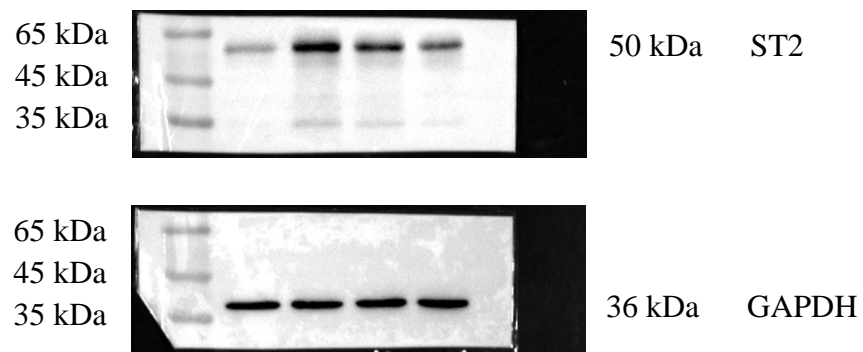

Repeat 3

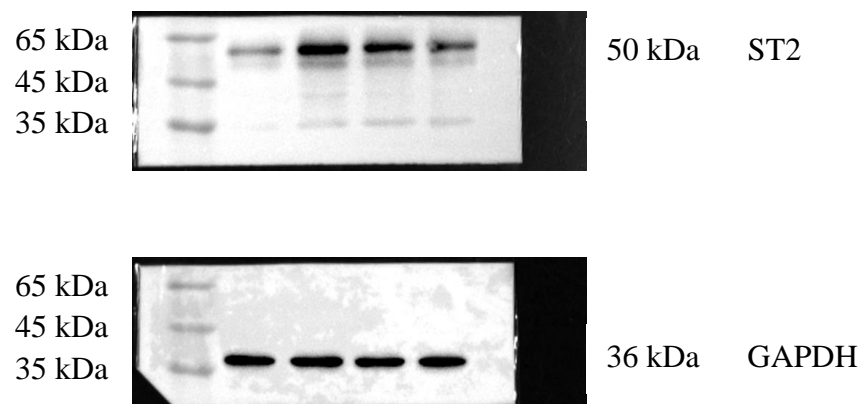

Supplement: Supplementary file 2 — Supplementary Material 2 [file 40360_2024_751_MOESM2_ESM.pdf]
